# Comparison of Mechanical Properties of Patient-Specific Direct 3D-Printed Aortic Valve for Simulation Trainings: A Comparative Study

**DOI:** 10.1177/15569845251408924

**Published:** 2026-02-11

**Authors:** Shokoufeh Cheheili Sobbi, Anastasiia Pavlykova-Chertovska, Silke Dreesen, Jos Maesen, Peyman Sardari Nia

**Affiliations:** 1 Department of Cardiothoracic Surgery, Heart and Vascular Centre, Maastricht University Medical Centre, The Netherlands; 2 Maastricht University, The Netherlands; 3 Biomedical Engineering Group of Department of Cardiology, Erasmus Medical Centre, Rotterdam, The Netherlands

**Keywords:** aortic valve surgery, endoscopic aortic valve replacement, endoscopic mitral valve surgery, simulation, telesimulation, 3-dimensional printing

## Abstract

**Objective:**

This study aims to evaluate the mechanical properties of 3-dimensional (3D)–printed patient-specific aortic models for surgical simulation. The objective was to analyze the effects of postcuring time and color pigments on material stiffness, flexibility, toughness, and durability and to identify the most suitable material and processing conditions to enhance the biomechanical realism of 3D-printed aortae.

**Methods:**

Direct 3D printing was used to fabricate patient-specific aortic models. Samples were prepared with different postcuring times (5, 10, 15, and 20 min) and with or without color pigments (pink and orange). Uniaxial tensile testing was conducted to analyze the mechanical properties, including tensile strength, stiffness, flexibility, and strain at failure, using stress–strain curves.

**Results:**

Uncolored samples with a postcuring time of 15 min demonstrated the best mechanical properties for simulation training, offering a balance between flexibility, stiffness, and toughness. Colored samples exhibited lower tensile strength, reduced toughness, and increased stiffness as compared with uncolored samples.

**Conclusions:**

Material selection and postprocessing play a crucial role in the biomechanical accuracy of 3D-printed patient-specific aortic models. Uncolored samples with a 15 min postcuring time are optimal for surgical simulation. Future research should focus on refining postprocessing techniques and directly comparing 3D-printed models with human aortic tissue to improve realism and validation.

## Graphical abstract

**Graphical Abstract:**
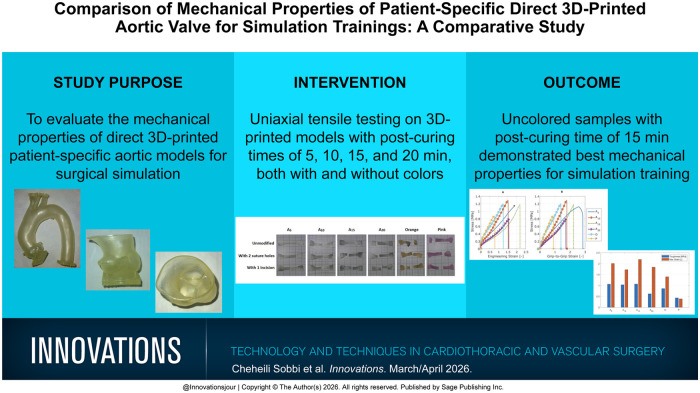
This is a visual representation of the abstract.

**Central Message**

We analyzed the material properties of 3D-printed patient-specific aortae, including the aortic valve, intended for simulation training, focusing on stiffness, flexibility, and reusability. The analysis found that the optimal simulation samples were uncolored with a 15 min postcuring time.

## Introduction

Three-dimensional (3D) printing has revolutionized surgical planning and simulation training in the past decade, offering highly detailed and patient-specific anatomical models for various surgical fields, including cardiac surgery.^1^ In surgical training, 3D-printed models are used in various simulation platforms and offer a realistic, hands-on learning experience, allowing trainees to practice procedures in a controlled, risk-free environment. In cardiac surgery, for example, patient-specific 3D-printed heart valves and vascular structures are used to refine surgical techniques, optimize device sizing, and improve perioperative planning.^2^ The high-fidelity minimally invasive mitral valve surgery simulator and telesimulator for endoscopic mitral valve surgery developed by Sardari Nia et al. have proven to be effective tools for simulation-based training.^3,4^ In our previous research, we developed a cost-effective method for directly 3D printing patient-specific mitral valves using soft materials, specifically designed for simulation-based training and procedural planning.^5^ A crucial factor in the effectiveness of 3D-printed anatomical models for surgical training is their material properties, which must accurately replicate the stiffness and flexibility of real tissues as well as ensure durability for simulation training. The mechanical properties of these models play a crucial role in providing a realistic simulation experience, allowing surgeons to practice techniques such as suturing, cutting, and deploying implants under conditions that closely resemble real-life surgery.^6^

This study examines the material properties of 3D-printed models intended for simulation training, focusing on stiffness, flexibility, and reusability.

## Methods

### Direct 3D Printing

**Fig. 1.**
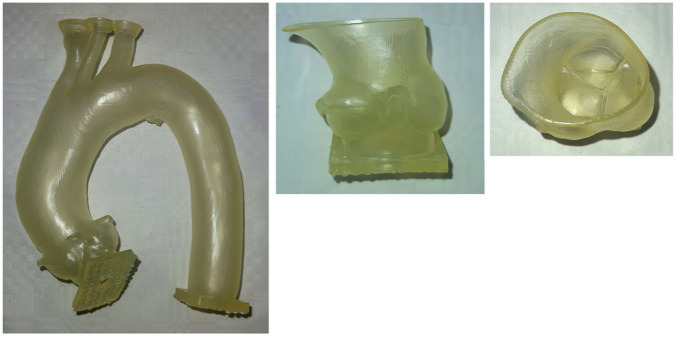
Different views of direct 3-dimensional–printed patient-specific aortae including the aortic valve.

The direct 3D printing of patient-specific aortae, including aortic valve (Fig. 1), followed the same process outlined in our previous research.^5^ This process includes the following:

- Data acquisition using 3D transesophageal echocardiography and computed tomography in Cartesian Digital Imaging and Communication in Medicine (DICOM) format
- Image processing with software programs such as Vesalius3D, Blender, MeshLab, and Atum3D Operation Station
- The 3D printing using digital light processing, an additive manufacturing technique based on photopolymer resins

### Material Analysis

We conducted experiments on 3D-printed models with different postcuring times of 5, 10, 15, and 20 min as well as on models with and without color, using pink and orange pigments. Both of the colored 3D-printed models had a postcuring time of 10 min.

For each type of 3D print, 3 samples were tested: 1 with a small incision in the middle to simulate cutting conditions, 1 with 2 suture holes in the middle to simulate suturing conditions, and 1 without modifications.

Uniaxial tensile testing was conducted for material analysis using the BioTester 5000 uniaxial test system (CellScale, Waterloo, ON, Canada). Uniaxial tensile testing is one of the most widely used methods for assessing the tensile properties of materials by stretching them in a single direction and measuring the resulting stress until failure.^7^

The 3D-printed models were cut into “dogbone” samples, with all cuts made at the same location and with the same thickness to ensure homogeneity. The samples were then speckled with matte black quick-drying spray paint applied through a mesh screen, with a ratio of 50:50, and dried for 1 min at room temperature. Next, the samples were clamped at both ends and loaded into the BioTester 5000. Each sample was straightened by applying a prestretch of 0.05 N to eliminate slack before collection of load data and calculation of strain. The samples were then stretched at a controlled rate in a horizontal direction according to the manufacturer’s protocol and as published in earlier research.^8,9^ The applied force (mN) and the resulting deformation (µm) in the middle part of the samples were continuously measured using image-tracking analysis with a high-speed camera positioned above the tensile-testing system. The image acquisition software captured at 30 frames per second with a resolution of 5.2 MP.

Outputs from the tensile testing were collected to generate stress–strain curves that characterize the material’s response to tensile loading, including stiffness, strength, elasticity, and failure behavior.^10–12^ These parameters are explained below.

#### Stress

Stress is the internal force per unit area within a material resulting from an applied external load. Hereby the original cross-sectional area was assumed to be rectangular and determined by multiplying the sample width by its thickness (equation 1).

(1) $$\sigma =F/A_{0}=F/w.t$$

where

-
σ = stress (Pa or N/m^2^)
- F = applied force (N)
-
$A_{0}$ = original cross-sectional area of the material (m^2^)
- *w* = width (m)
- *t* = thickness (m)

#### Engineering strain

Strain is a measure of a material’s deformation under applied stress. It is a dimensionless quantity representing the relative change in length. Engineering strain was calculated according to equation 2.

(2) $$\varepsilon ==\Delta L/L_{0}=(L-L_{0})/L_{0}$$

where

-
ε = engineering strain (unitless)
-
DL = change in length (m)
-
$L_{0}$ = original length (m)
-
L = final length (m)

#### Grip-to-grip strain

Grip-to-grip strain is the strain measured based on the distance between the grips in a tensile-testing setup. Rather than focusing on the material itself, it is determined by tracking the displacement of the grips securing the sample and is calculated according to equation 3.

(3) $$\varepsilon_{grip}==\Delta L_{grip}/L_{0,grip}=(L_{grip}-L_{0,grip})/L_{0,grip}$$

where

-
$\varepsilon_{grip}$ = grip-to-grip strain (unitless)
-
$DL_{grip}$ = change in grip-to-grip length (m)
-
$L_{0,grip}$ = original grip-to-grip length (m)
-
$L_{grip}$ = final grip-to-grip length (m)

#### Stress–strain curve

**Fig. 2.**
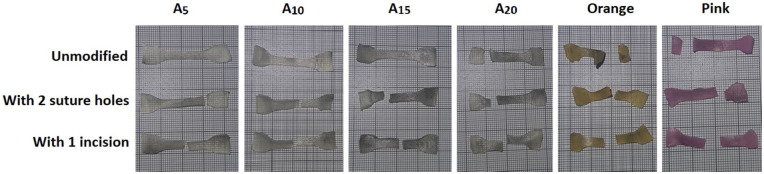
Schematic illustration of the stress–strain curve with elastic, plastic, and necking regions. Yield, ultimate tensile strength, and fracture points are shown.

After calculation, the stress–strain curves for each sample were plotted using MATLAB (R2022b, The MathWorks, Natick, MA, USA). The graph’s coordinate axes are defined with strain on the *x*-axis and stress on the *y*-axis. The elastic modulus, ultimate tensile strength, and strain at failure were extracted from the stress–strain curve, as schematically illustrated in Figure 2.

The graphical quality of the stress–strain curves was evaluated after plotting:

- Elastic region: the material deforms but returns to its original shape when the force is removed
- Yield point: marks the transition from elastic to plastic deformation; beyond this point, the material will not return to its original shape
- Plastic region: the material undergoes permanent deformation; stress continues to increase until it reaches the ultimate tensile strength, the maximum stress the material can withstand
- Necking region and fracture point: at a certain strain level, the material fractures or breaks; this marks the failure point on the stress–strain curve

### Data Analysis

Tensile properties from the stress–strain curves were collated within a master spreadsheet using Excel version 2210 (Microsoft, Redmond, WA, USA). Scatter and bar charts were created in MATLAB.

## Results

**Fig. 3.**
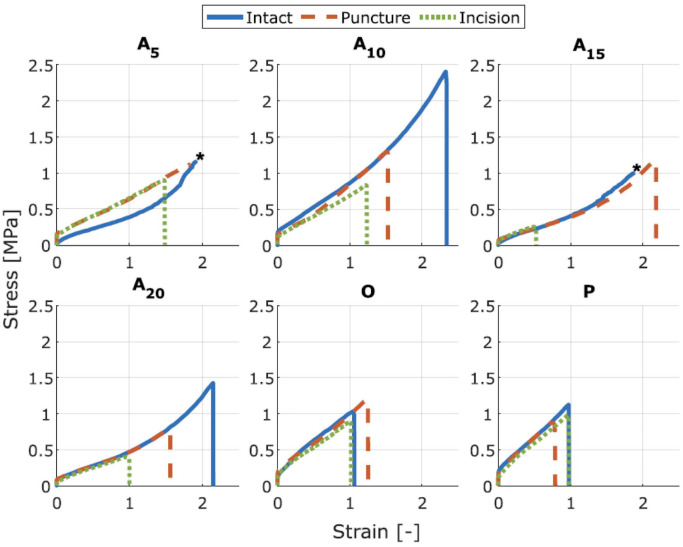
Pictures of all samples after the tensile testing. The A5 samples had postcuring times of 5 min. The A10 samples had postcuring times of 10 min. The A15 samples had postcuring times of 15 min. The A20 samples had postcuring times of 20 min. The O samples were colored with orange pigments and had postcuring times of 10 min. The P samples were colored with pink pigments and had postcuring times of 10 min.

**Fig. 4.**
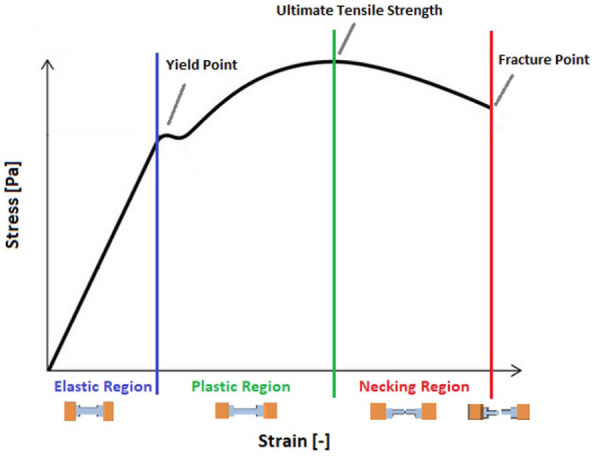
Stress–strain graphs of all the samples. The A5 samples had postcuring times of 5 min. The A10 samples had postcuring times of 10 min. The A15 samples had postcuring times of 15 min. The A20 samples had postcuring times of 20 min. The O samples were colored with orange pigments and had postcuring times of 10 min. The P samples were colored with pink pigments and had postcuring times of 10 min.

A total of 18 samples were analyzed (Fig. 3, Fig. 4). Several factors may have influenced the results. Both the unmodified sample with a postcuring time of 5 min (A5) and the sample with a postcuring time of 15 min (A15) slipped out of the grips, and A15 with 2 suture holes slipped again in the second test. This suggests that the recorded maximum stress and strain values may be lower than their actual values.

For the incision samples, ensuring a consistent cut size is challenging, making it impossible to perform a precise analysis of maximum strain or stress. Because the depth of the incision strongly influences the strain values, absolute comparisons between samples in the incision test are not valid. In addition, in the incision test for A15, a significantly larger cut was made compared with the other samples, further reducing the maximum stress and strain values.

**Fig. 5.**
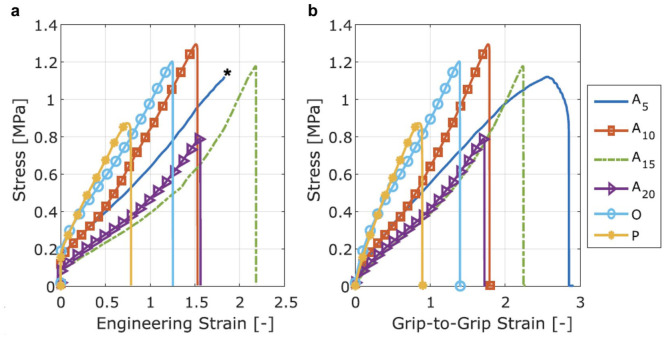
Stress–strain graphs for the samples with 2 suture holes in the middle. (a) The stress data are plotted against engineering strain. (b) The stress data are plotted against grip-to-grip displacement.

For the samples with 2 suture holes, the stress-engineering strain graph and the stress data plotted against grip-to-grip displacement are shown in Figure 5. The strain was calculated at the center of the sample, between the 2 holes. Each sample eventually ruptured, except for A5, where the curve stopped because the sample slipped out of the grips.

**Fig. 6.**
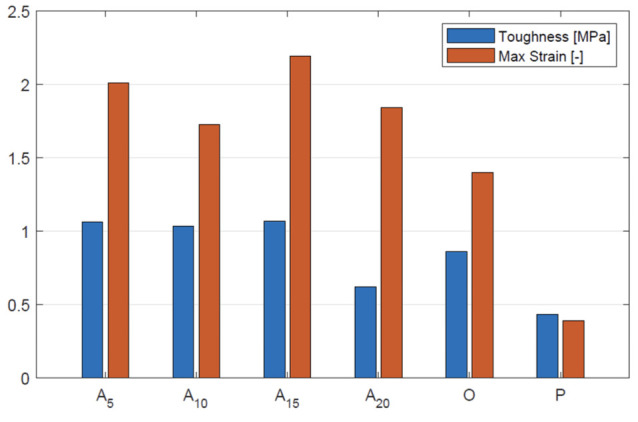
Bar plots for toughness and flexibility of the samples with 2 suture holes in the middle. The energy (MPa) the material can absorb before failure is shown in the blue bars. The maximum strain just next to the suture hole is shown in the orange bars.

The toughness of the material after puncture was determined from the stress-engineering strain graph (Fig. 5a), calculated as the area under the curve. This represents the energy the material can absorb before failure. These results are displayed in the bar plot (blue bars) in Figure 6. Samples A5, A10, and A15 exhibited similar toughness, whereas the other samples showed lower values. It is important to note that A5 did not rupture but slipped out of the grips, meaning its actual toughness is likely slightly higher.

In addition to toughness, the maximum strain just next to the hole was also analyzed and is shown in the bar plot (orange bars) in Figure 6. Since both toughness and flexibility are relevant, this metric provides additional insight. Given that A5 did not rupture, its actual maximum strain is likely slightly higher.

Finally, the stiffness of the materials can be compared using the stress-engineering strain graph (Fig. 5a). A5 and A10 are slightly stiffer than A15 and A20, which aligns with expectations.

## Discussion

The findings from this study provide valuable insights into the mechanical properties of 3D-printed patient-specific aortae intended for surgical simulation. The uniaxial tensile-testing results demonstrated variations in stiffness, toughness, flexibility, and strain at failure, which are critical parameters for assessing the biomechanical fidelity of these models. However, several factors must be considered when interpreting these results, particularly regarding the sample preparation and testing limitations.

One of the challenges encountered was sample slippage during testing, particularly in unmodified A5 and unmodified A15, which led to premature termination of the stress–strain curves. This indicates that the maximum stress and strain values recorded for these samples may not reflect their true mechanical capacity. Sample slippage is a well-documented limitation in uniaxial tensile testing, particularly when testing soft materials with low surface friction and high compliance.^13^ Future studies may consider modifying the grip design or surface treatment to minimize this issue and improve measurement accuracy.^14^

For the incision samples, variations in cut size introduced additional variability, making it difficult to establish absolute comparisons in terms of maximum strain and stress. The depth of the incision significantly affects localized stress distribution, and inconsistencies in cut dimensions may have led to lower recorded values for A15 containing an incision. This highlights the importance of standardized incision protocols in mechanical testing to ensure reproducibility. However, the results of this study demonstrate that an incision weakens the material significantly more than punctures.

Given these limitations and the primary focus on suturing in simulation training, a detailed analysis was conducted on the samples with 2 suture holes, as explained below.

Comparing stiffness across samples, A5 and A10 were found to be slightly stiffer than A15 and A20. These findings align with previous studies that have reported postcuring time as a important factor influencing the mechanical properties of 3D-printed materials.^15^ Longer postcuring durations typically lead to increased polymer crosslinking, thereby enhancing stiffness but potentially reducing flexibility.^16^ The results of our study support these observations and indicate that careful tuning of postcuring conditions is necessary to achieve an optimal balance between stiffness and elasticity.

In the context of simulation training, an ideal material must not only possess the appropriate stiffness and flexibility but also maintain durability for repeated use. The results suggest that materials with moderate postcuring times (15 min) may offer an optimal balance, as they exhibit sufficient toughness and flexibility while maintaining structural integrity.

In addition, although adding color pigments allows for better visualization of 3D-printed aortae, colored samples showed lower tensile strength, increased stiffness, reduced toughness, and decreased flexibility and elasticity compared with the translucent samples. Earlier studies have demonstrated that pigments can alter the polymer matrix structure. Depending on their size and distribution, they may either reinforce the material or introduce weak points.^17–19^ In addition, pigments can interfere with polymer bonding, leading to weaker interlayer adhesion in fused deposition modeling and reduced crosslinking in resin-based prints.^17–19^ Moreover, pigments may affect polymerization depth, potentially resulting in incomplete curing.^17–19^

Another limitation of our study is the lack of direct comparison with human aortic tissue. Evaluating the mechanical properties of 3D-printed models against real human aortae would provide a more accurate assessment of their suitability for surgical simulation.

Moreover, the materials were not yet evaluated by surgeons in a clinical simulation context. This aspect is important for assessing the clinical relevance and realism of the models and should be addressed in future studies to better understand how closely the simulated materials approximate real tissue properties and user experience.

## Conclusions

This study highlights the importance of material selection and postprocessing techniques in developing high-fidelity 3D-printed patient-specific aortae for surgical simulation. Our findings indicate that uncolored samples with a postcuring time of 15 min exhibit the most suitable mechanical properties for simulation training. To further enhance the biomechanical accuracy of these models, future research should focus on refining postprocessing methods and optimizing material selection. In addition, a thorough evaluation of pigmented materials is necessary to ensure they closely replicate the mechanical behavior of real tissue. To validate the realism of 3D-printed aortae, future studies should incorporate biomechanical testing that directly compares 3D-printed samples with fresh human aortic tissue as well as evaluation by surgeons to further assess the clinical relevance and realism of the models.
